# Supplementary material for: A rechargeable Ca/Cl2 battery
Source: Nat Commun. 2024 Jan 31;15:944. doi: 10.1038/s41467-024-45347-3 (PMC10831116; doi:10.1038/s41467-024-45347-3)
Supplement: Supplementary file 1 — Reporting Summary [file 41467_2024_45347_MOESM1_ESM.pdf]

nature portfolio

Corresponding author(s): Hao Sun

Last updated by author(s): Jan. 6, 2024

Reporting Summary

Nature Portfolio wishes to improve the reproducibility of the work that we publish. This form provides structure for consistency and transparency in reporting. For further information on Nature Portfolio policies, see our Editorial Policies and the Editorial Policy Checklist.

Statistics

For all statistical analyses, confirm that the following items are present in the figure legend, table legend, main text, or Methods section.

n/a	Confirmed	
☐	☒	The exact sample size (*n*) for each experimental group/condition, given as a discrete number and unit of measurement
☐	☒	A statement on whether measurements were taken from distinct samples or whether the same sample was measured repeatedly
☒	☐	The statistical test(s) used AND whether they are one- or two-sided<br>*Only common tests should be described solely by name; describe more complex techniques in the Methods section.*
☒	☐	A description of all covariates tested
☒	☐	A description of any assumptions or corrections, such as tests of normality and adjustment for multiple comparisons
☐	☒	A full description of the statistical parameters including central tendency (e.g. means) or other basic estimates (e.g. regression coefficient) AND variation (e.g. standard deviation) or associated estimates of uncertainty (e.g. confidence intervals)
☒	☐	For null hypothesis testing, the test statistic (e.g. *F*, *t*, *r*) with confidence intervals, effect sizes, degrees of freedom and *P* value noted<br>*Give P values as exact values whenever suitable.*
☒	☐	For Bayesian analysis, information on the choice of priors and Markov chain Monte Carlo settings
☒	☐	For hierarchical and complex designs, identification of the appropriate level for tests and full reporting of outcomes
☒	☐	Estimates of effect sizes (e.g. Cohen's *d*, Pearson's *r*), indicating how they were calculated

Our web collection on statistics for biologists contains articles on many of the points above.

Software and code

Policy information about availability of computer code

Data collection	X-ray diffraction patterns were acquired from a Bruker D8 Advance powder X-ray diffractometer with Cu Ka (λ = 0.15406 nm). Scanning electron microscopy and transmission electron microscopy were performed on a ZEISS Gemini 300 and JEOL JEM-2100F, respectively. Time-of-flight secondary ion mass spectrometry was performed on an ION-TOF 5-100 (ION-TOF GmbH). A Thermo ESCALAB 250Xl was used for X-ray photoelectron spectroscopy analyses. Raman spectra were obtained using a HeNe laser (532 nm). Differential electrochemical mass spectrometry was carried out using a commercial quadrupole mass spectrometer (Linglu Instrument). A FEI Talos F200X G2 microscope was used for cryogenic transmission electron microscopy (cryo-TEM) characterization under 200 kV. Thermogravimetric analysis (TGA) was performed using PerkinElmer TGA 550. Differential Scanning Calorimetry (DSC) was performed on a DSC-250 equipment. Ca K-edge X-ray absorption spectra (XAS) were carried out at 4B7A station of Beijing Synchrotron Radiation Facility (BSRF). The ion conductivities of electrolytes were measured using a FE38 conductivity meter of FiveEasy Plus.
Data analysis	Origin, photoshop, Athena

For manuscripts utilizing custom algorithms or software that are central to the research but not yet described in published literature, software must be made available to editors and reviewers. We strongly encourage code deposition in a community repository (e.g. GitHub). See the Nature Portfolio guidelines for submitting code & software for further information.

Data

Policy information about availability of data

All manuscripts must include a data availability statement. This statement should provide the following information, where applicable:

- Accession codes, unique identifiers, or web links for publicly available datasets
- A description of any restrictions on data availability
- For clinical datasets or third party data, please ensure that the statement adheres to our policy

The data that support the findings of this study are available within the text including the Methods and Supplemental information. Raw datasets related to the current work are available from the corresponding author on reasonable request.

Research involving human participants, their data, or biological material

Policy information about studies with human participants or human data. See also policy information about sex, gender (identity/presentation), and sexual orientation and race, ethnicity and racism.

Reporting on sex and gender	The findings apply to all sex and gender. No sex and gender were considered in study design.
Reporting on race, ethnicity, or other socially relevant groupings	There are no data about the race, ethnicity, or other socially relevant groupings in our research.
Population characteristics	*Describe the covariate-relevant population characteristics of the human research participants (e.g. age, genotypic information, past and current diagnosis and treatment categories). If you filled out the behavioural & social sciences study design questions and have nothing to add here, write "See above."*
Recruitment	*Describe how participants were recruited. Outline any potential self-selection bias or other biases that may be present and how these are likely to impact results.*
Ethics oversight	*Identify the organization(s) that approved the study protocol.*

Note that full information on the approval of the study protocol must also be provided in the manuscript.

Field-specific reporting

Please select the one below that is the best fit for your research. If you are not sure, read the appropriate sections before making your selection.

☐ Life sciences ☐ Behavioural & social sciences ☒ Ecological, evolutionary & environmental sciences

For a reference copy of the document with all sections, see nature.com/documents/nr-reporting-summary-flat.pdf

Ecological, evolutionary & environmental sciences study design

All studies must disclose on these points even when the disclosure is negative.

Study description	Rechargeable Ca/Cl2 battery
Research sample	Calcium metal, graphite, thionyl chloride (SOCl2), aluminium chloride (AlCl3), lithium difluoro(oxalate)borate (LiDFOB)
Sampling strategy	Graphite was mixed with polytetrafluoroethylene (PTFE) at a mass ratio of 9:1 in ethanol solution. The obtained suspension was then subjected to ultrasonic treatment for 1h to obtain the cathode slurry. A Ni foam was cut into a 14 mm diameter (1.54 cm2) circular using a manual disk cutter (MSK-T-10, MTI), followed by dropping 50 µL cathode slurry onto it.<br><br>All the electrolytes were also prepared in the same glovebox. AlCl3 and SOCl2 were used as received. LiDFOB and CaCl2 were dried at 120 °C for 12 h in a vacuum chamber before use. Typically, 6 M AlCl3 and 1.3 M LiDFOB were dissolved in 1 mL SOCl2, followed by addition of 1.2 M CaCl2 under continuous stirring for 1 h to obtain the CALS electrolyte. The gelation of the CALS electrolyte generally took 2 h, which allowed us to assemble batteries using the electrolyte in liquid state.
Data collection	X-ray diffraction patterns were acquired from a Bruker D8 Advance powder X-ray diffractometer with Cu Ka (λ = 0.15406 nm). Scanning electron microscopy and transmission electron microscopy were performed on a ZEISS Gemini 300 and JEOL JEM-2100F, respectively. Time-of-flight secondary ion mass spectrometry was performed on an ION-TOF 5-100 (ION-TOF GmbH). A Thermo ESCALAB 250Xl was used for X-ray photoelectron spectroscopy analyses. Raman spectra were obtained using a HeNe laser (532 nm). Differential electrochemical mass spectrometry was carried out using a commercial quadrupole mass spectrometer (Linglu Instrument). A FEI Talos F200X G2 microscope was used for cryogenic transmission electron microscopy (cryo-TEM) characterization under 200 kV. Thermogravimetric analysis (TGA) was performed using PerkinElmer TGA 550. Differential Scanning Calorimetry (DSC) was performed on a DSC-250 equipment. Ca K-edge X-ray absorption spectra (XAS) were carried out at 4B7A station of Beijing

Synchrotron Radiation Facility (BSRF). The ion conductivities of electrolytes were measured using a FE38 conductivity meter of FiveEasy Plus.

Timing and spatial scale: Sep. 2021 - May 2023

Data exclusions: No data were excluded.

Reproducibility: All attempts to repeat the experiment were successful.

Randomization: *Describe how samples/organisms/participants were allocated into groups. If allocation was not random, describe how covariates were controlled. If this is not relevant to your study, explain why.*

Blinding: *Describe the extent of blinding used during data acquisition and analysis. If blinding was not possible, describe why OR explain why blinding was not relevant to your study.*

Did the study involve field work? ☐ Yes ☒ No

Reporting for specific materials, systems and methods

We require information from authors about some types of materials, experimental systems and methods used in many studies. Here, indicate whether each material, system or method listed is relevant to your study. If you are not sure if a list item applies to your research, read the appropriate section before selecting a response.

Materials & experimental systems

n/a	Involved in the study
☒	☐ Antibodies
☒	☐ Eukaryotic cell lines
☒	☐ Palaeontology and archaeology
☒	☐ Animals and other organisms
☒	☐ Clinical data
☒	☐ Dual use research of concern
☒	☐ Plants

Methods

n/a	Involved in the study
☒	☐ ChIP-seq
☒	☐ Flow cytometry
☒	☐ MRI-based neuroimaging

Plants

Seed stocks: *Report on the source of all seed stocks or other plant material used. If applicable, state the seed stock centre and catalogue number. If plant specimens were collected from the field, describe the collection location, date and sampling procedures.*

Novel plant genotypes: *Describe the methods by which all novel plant genotypes were produced. This includes those generated by transgenic approaches, gene editing, chemical/radiation-based mutagenesis and hybridization. For transgenic lines, describe the transformation method, the number of independent lines analyzed and the generation upon which experiments were performed. For gene-edited lines, describe the editor used, the endogenous sequence targeted for editing, the targeting guide RNA sequence (if applicable) and how the editor was applied.*

Authentication: *Describe any authentication procedures for each seed stock used or novel genotype generated. Describe any experiments used to assess the effect of a mutation and, where applicable, how potential secondary effects (e.g. second site T-DNA insertions, mosiacism, off-target gene editing) were examined.*
