# Supplementary material for: A rechargeable Ca/Cl2 battery
Source: Nat Commun. 2024 Jan 31;15:944. doi: 10.1038/s41467-024-45347-3 (PMC10831116; doi:10.1038/s41467-024-45347-3)
Supplement: Supplementary file 2 — Supplementary Information [file 41467_2024_45347_MOESM2_ESM.pdf]

Supplementary Information

A rechargeable Ca/Cl_2 battery

*Shitao Geng[1†], Xiaoju Zhao[1†], Qiuchen Xu[1], Bin Yuan[1], Yan Wang[1], Meng Liao[2], Lei Ye[2], Shuo Wang[1], Zhaofeng Ouyang[1], Liang Wu[1], Yongyang Wang[3], Chenyan Ma[3], Xiaojuan Zhao[3], Hao Sun[1]**

[1]Frontiers Science Center for Transformative Molecules, School of Chemistry and Chemical Engineering, and Zhangjiang Institute for Advanced Study, Shanghai Jiao Tong University, Shanghai 200240, China.

[2]Department of Mechanical Engineering, The Pennsylvania State University, University Park, PA 16802, USA.

[3]Beijing Synchrotron Radiation Facility (BSRF), Institute of High Energy Physics, Chinese Academy of Sciences, Beijing 100049, China.

†These authors contributed equally to this work.

**Correspondence and requests for materials should be addressed to Hao Sun (haosun@sjtu.edu.cn) (H.S.).*

Supplementary Methods

Characterization

X-ray diffraction patterns were acquired from a Bruker D8 Advance powder X-ray diffractometer with Cu Ka (λ = 0.15406 nm). Scanning electron microscopy and transmission electron microscopy were performed on a ZEISS Gemini 300 and JEOL JEM-2100F, respectively. Time-of-flight secondary ion mass spectrometry was performed on an ION-TOF 5-100 (ION-TOF GmbH) under the pressure of the analysis chamber below 1.1×10^{-9} mbar. The organic imaging with delay extraction mode with pulsed 30 keV Bi^{3+} (0.16 to 0.28 pA pulsed current) ion beam was applied for depth profiling (< 800 nm) analysis, and the typical analysis area was 80 ×80 μm^2, with the Cs^+ ion beam of 2 keV sputtering at the same time (69.27 to 82.74 nA current), 260 μm × 260 μm sputter raster. A Thermo ESCALAB 250Xl was used for X-ray photoelectron spectroscopy analyses with a monochromatic Al $K\alpha$ source (hv = 1486.6 eV) and a charge neutralization system. The XPS depth profiling was performed using a monatomic Ar ion etching source. The energy range was 2 keV. The vacuum degree of the analysis chamber was less than 5×10^{-9} Torr. All the samples were transferred to the inlet chamber through an air-isolated chamber. All the binding energies were calibrated with the C1s peak (284.8 eV). For Raman analysis, the electrolytes were sealed in a transparent capillary tube without Raman signals. Raman spectra (200–2,000 cm^{-1}) were obtained using a HeNe laser (532 nm). Differential electrochemical mass spectrometry was carried out using a commercial quadrupole mass spectrometer (Linglu Instrument) and a custom designed Swagelok battery equipped with two poly(ether-ether-ketone) capillary tubes as the gas inlet and outlet. A graphite cathode with a mass loading of 4 mg cm^{-2} was used for cell preparation. The tested cell was first flushed with Ar gas at a constant flow rate of 1 mL min^{-1} for 6 h to calibrate the baseline, and was then discharged and charged at 300 mA g^{-1} for product analysis. A FEI Talos F200X G2 microscope was used for cryogenic transmission electron microscopy (cryo-TEM) characterization under 200 kV. A Cu mesh loaded sample was transferred to the cryo-holder (Fischione 2550) using an Ar-filled glove box, and then the sample holder was quickly inserted inside the microscope. Liquid nitrogen was poured into the cryo-holder and maintained the sample temperature at about −170 °C. Thermogravimetric analysis (TGA) was performed using PerkinElmer TGA 550. The sample was heated from 30 to 400 °C at a heating rate of 5°C min^{-1}. Differential Scanning Calorimetry (DSC) was performed on a DSC−250 equipment at a heating rate of 5 °C min^{-1} in the temperature range from −70 °C to 45 °C under

an N_2 atmosphere. Ca *K*-edge X-ray absorption spectra (XAS) were carried out at 4B7A station of Beijing Synchrotron Radiation Facility (BSRF). The beamline was operated at 2.5 GeV with 250 mA maximum current using a Si (111) double-crystal monochromator. The spectra of $CaCl_2$ was used as standard and measured at a total electron yield mode at a vacuum degree of 10^{-3} to 10^{-4} Pa. The liquid and gel electrolyte samples were measured at the fluorescence mode under helium atmosphere. All the XANES (X-ray Absorption Near Edge Structure) and EXAFS (Extended X-ray Absorption Fine Structure) spectra were aligned, normalized and merged using Athena software. The ion conductivities of electrolytes were measured using a FE38 conductivity meter of FiveEasy Plus after calibration.

Molecular dynamics (MD) simulations

The chemical species in the simulated system were $AlCl_4^-$, $DFOB^-$, Li^+, Ca^{2+}, Cl^-, $SOCl^+$, $SOCl_2$, and their composition was determined by the actual concentration of each species in the electrolyte. In particular, $AlCl_3$ could react with equal molar amount of $SOCl_2$ to form $SOCl^+$ and $AlCl_4^-$ according to the following equation[1], which resulted in the coexistence of $SOCl_2$ and $SOCl^+$ in our simulated system:

$$SOCl_2 + AlCl_3 \rightarrow SOCl^+ + AlCl_4^- \qquad (S1)$$

Therefore, the electrolyte composition of the 6 M $AlCl_3$, 1.2 M $CaCl_2$ and 1.3 M LiDFOB in $SOCl_2$ electrolyte was 6 M $AlCl_4^-$ 1.2 M Ca^{2+}, 2.4 M Cl^-, 1.3 M Li^+, 1.3 M $DFOB^-$, 7.7 M $SOCl_2$ and 6 M $SOCl^+$. The simulation box for CALS electrolyte contained 1100 $AlCl_4^-$, 238 $DFOB^-$, 238 Li^+, 220 Ca^{2+}, 440 Cl^-, 1100 $SOCl^+$, and 1411 $SOCl_2$, respectively. As for CAS system, the ions and numbers were the same as CALS system except for the existence of $DFOB^-$ and Li^+ ions.

All MD simulations were conducted using the GROMACS 2019.3[2] to investigate the solvation structure of electrolyte. Parameters for $DFOB^-$, $SOCl_2$ and $SOCl^+$ were generated with the antechamber module of Amber18[3] using the general Amber force field (GAFF)[4], with partial charges set to fit the electrostatic potential generated with B3LYP/def2TZVP by the restrained electrostatic potential (RESP)[5]. Parameters for $AlCl_4^-$ were obtained from previous work[6]. The electrolyte was placed in a periodic cubic box, in which the NPT ensemble (constant number of particles, temperature, and pressure) was applied[7,8]. The temperature and pressure were coupled *via* V-rescale thermostat (298.15 K) and Parrinello−Rahman barostat (the reference pressure of 1 bar). The cutoff radius for the neighbor searching and nonbonded interactions

was taken to be 12 Å, and all the bonds were constrained using the LINCS algorithm[9]. The system was fully equilibrated after 100 ns simulation and another 100 ns was run to collect the date for statistical analysis. All the computed structures in MD simulations were illustrated using visual molecular dynamics (VMD)[10]. The radial pair distribution function $g(r)$ was also calculated through VMD. The electrolyte densities of CALS and CAS using the NPT ensemble were 1.047 and 0.952 g cm^{-3}, respectively. The long-range electrostatic interaction was calculated based on the Particle-mesh Ewald (PME) method[11]. It used Ewald summation to split up the calculation into a short-range part, for which all interactions are directly evaluated up to a cutoff radius r_c (1.2 nm), and a long-range part, which is solved in a reciprocal space. To take advantage of fast Fourier transforms (FFTs) for the conversions to reciprocal space, the charges are interpolated onto a uniform grid using cardinal B-splines.

Supplementary Figures

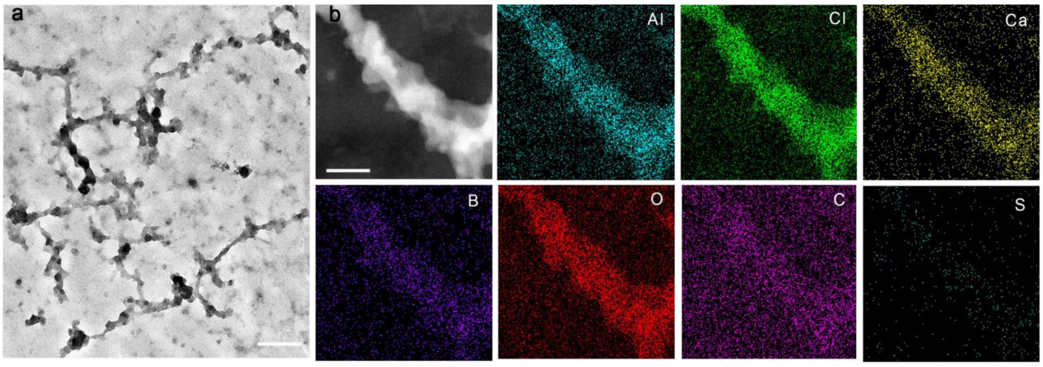

Supplementary Fig. 1 TEM and EDS images of the CALS electrolyte. a, TEM image of the CALS electrolyte. Scale bars, 500 nm. **b**, EDS images of the CALS electrolyte. Scale bars, 200 nm. Well-overlapped elements of Al, Cl, Ca, B, O, C and S indicate the uniform distribution of Al^{3+}, Ca^{2+}, Cl^{-} and $DFOB^{-}$. The CALS electrolyte was vacuumed for 1 h to remove the volatile components in prior to characterization.

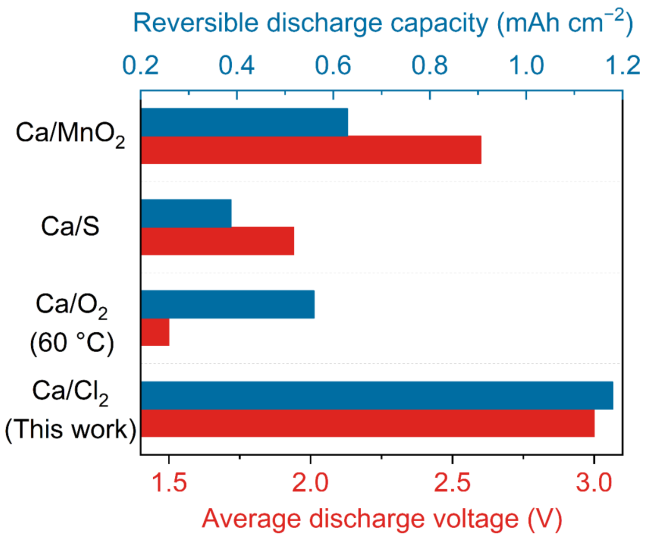

Supplementary Fig. 2 Electrochemical performance comparison of the rechargeable Ca/Cl_2 battery with the other Ca metal batteries. Comparison of the reversible areal capacity and discharge voltage of our rechargeable Ca/Cl_2 battery with other representative Ca metal batteries[12-14].

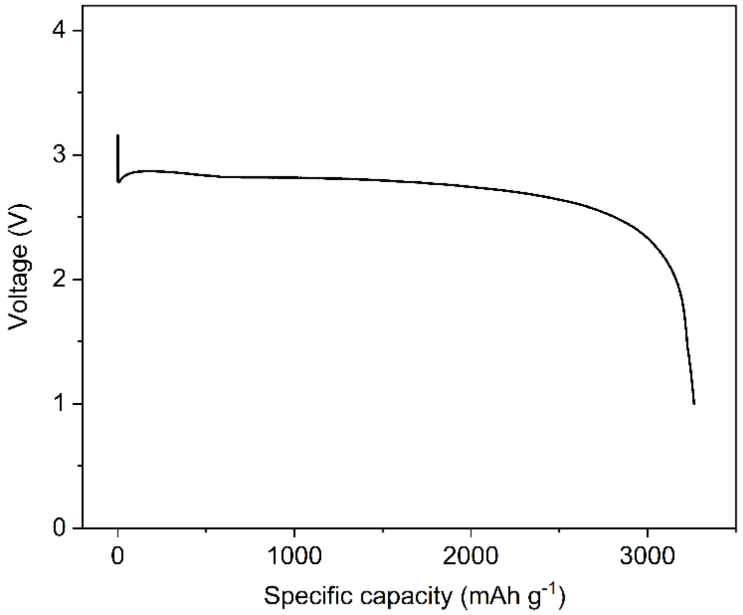

Supplementary Fig. 3 The initial discharge profile of a Ca/Cl_2 battery using the CALS electrolyte. The discharge capacity was 3,264 mAh g^{-1} with a distinct discharge plateau of 2.8 V *vs.* Ca/Ca^{2+} at 100 mA g^{-1}.

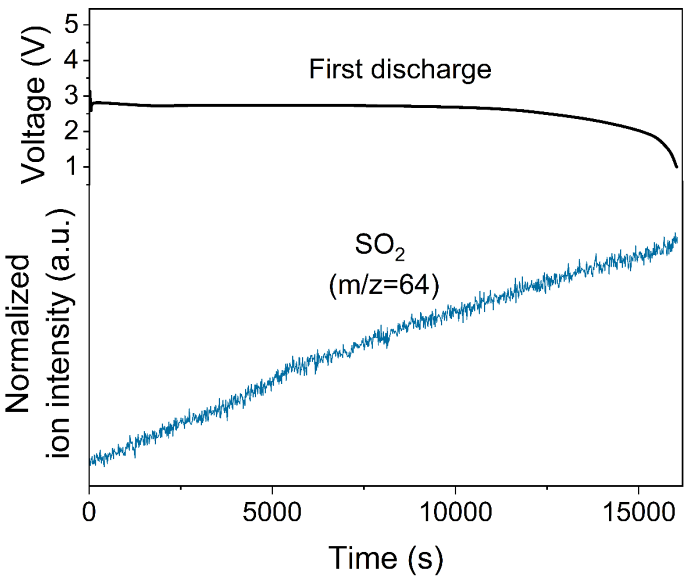

Supplementary Fig. 4 DEMS analysis of the rechargeable Ca/Cl_2 battery during the first discharge. Current density, 300 mA g^{-1}. Charge capacity, 450 mAh g^{-1}. Mass loading of graphite, 4 mg cm^{-2}.

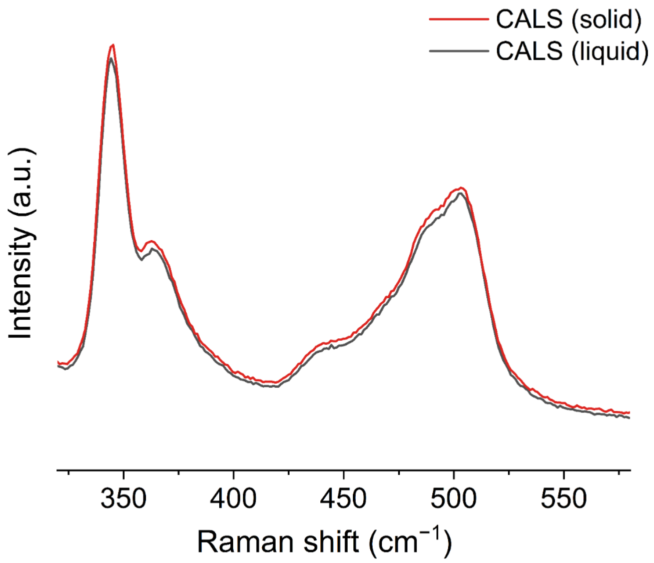

Supplementary Fig. 5 Raman spectra of the CALS electrolyte before and after gelation. The solvation structure of the CALS electrolyte remained almost unchanged before and after gelation.

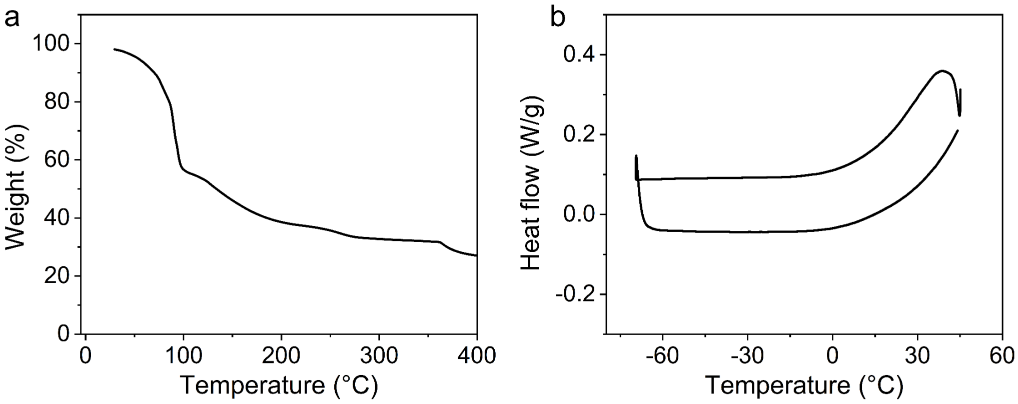

Supplementary Fig. 6. **TGA and DSC analysis of the CALS electrolyte**. **a**, TGA curve of the CALS electrolyte. **b**, DSC curve of the CALS electrolyte.

We performed thermogravimetric analysis (TGA) measurement of the CALS electrolyte, which excluded chemical bonding or reactions between the components in the electrolyte (**Supplementary Fig. 6a**). Differential scanning calorimetry (DSC) profiles showed no heat change or phase transition of the CALS electrolyte, indicating that the CALS electrolyte was a physical mixture (**Supplementary Fig. 6b**). Therefore, the gelation of the CALS electrolyte may attribute to the intermolecular interactions between different substances such as Ca^{2+} and $DFOB^{-}$.[15,16]

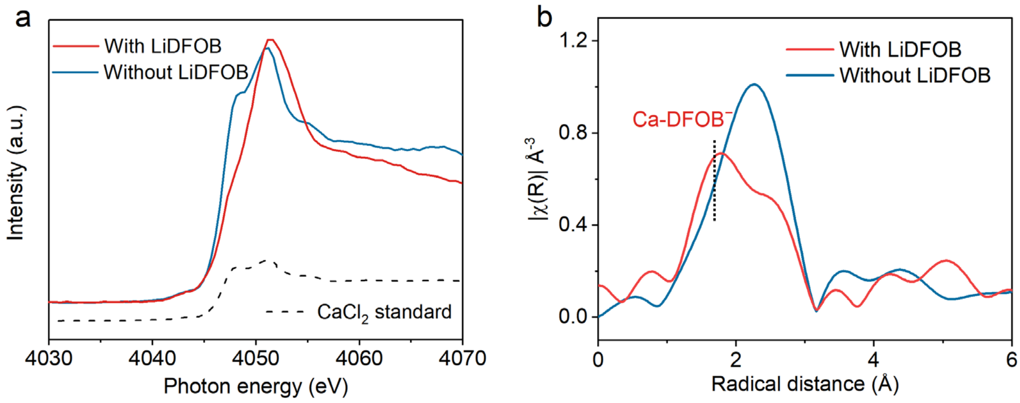

Supplementary Fig. 7 XAS profiles of the CALS electrolytes with and without LiDFOB. **a**, Ca *K*-edge XANES spectra of the $CaCl_2/AlCl_3/LiDFOB/SOCl_2$ and $CaCl_2/AlCl_3/SOCl_2$ electrolytes. **b**, Fourier-transformed magnitudes of the Ca *K*-edge EXAFS spectra of the $CaCl_2/AlCl_3/LiDFOB/SOCl_2$ and $CaCl_2/AlCl_3/SOCl_2$ electrolytes.

According to the Ca *K*-edge XANES spectra, the shoulder peak of Ca^{2+}–Cl^{-} in the first solvation shell (4048 eV) was largely weakened with $DFOB^{-}$ addition (**Supplementary Fig. 7a**). Meanwhile, a new Ca^{2+}–$DFOB^{-}$ coordinating pair with a shorter radical distance than that of pristine Ca^{2+}–Cl^{-} pair emerged based on Fourier-transformed magnitudes of the Ca *K*-edge EXAFS spectra (**Supplementary Fig. 7b**). This result confirmed the engagement of $DFOB^{-}$ ions inside the inner solvation shell of Ca^{2+} ions, which was consistent with the MD simulations and RDF results in **Fig. 2**.

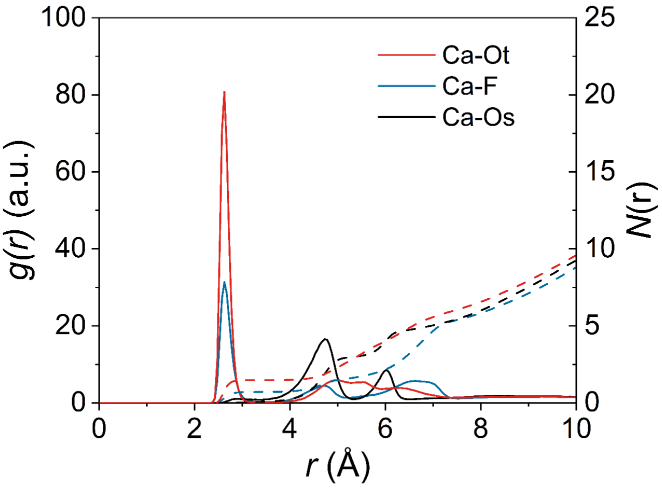

Supplementary Fig. 8 RDFs of Ca^{2+}–F, Ca^{2+}–O_s, and Ca^{2+}–O_t of $DFOB^{-}$. Note that O_s and O_t represent the shoulder O and tailed O in $DFOB^{-}$, respectively.

To elucidate the interaction preference inside a Ca^{2+}–$DFOB^{-}$ ion pair, we demonstrated the radical distribution functions (RDFs) of Ca^{2+}–F, Ca^{2+}–O_s, and Ca^{2+}–O_t respectively. As a result, the interaction intensity followed the order of Ca^{2+}–O_t > Ca^{2+}–F >> Ca^{2+}–O_s, validating that both tailed C=O and B−F bonds were engaged in the solvation shell of Ca^{2+} ions.

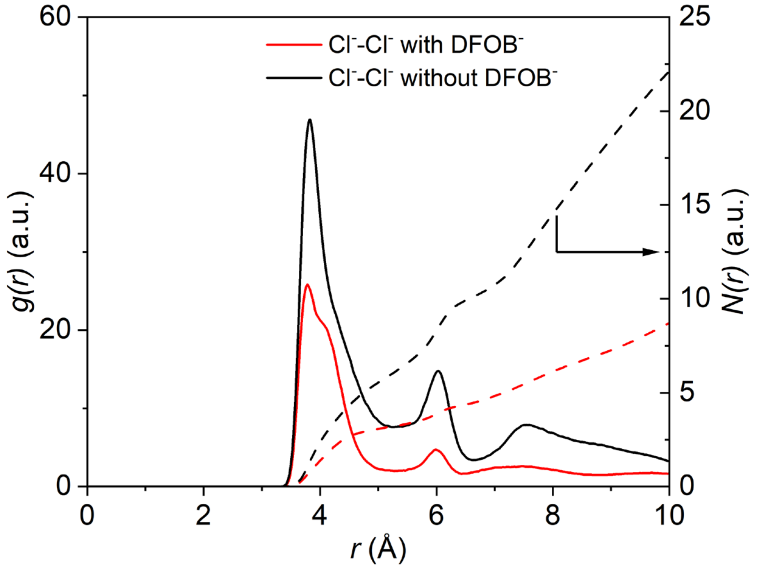

Supplementary Fig. 9 RDFs of Cl^-−Cl^- in CALS electrolyte with and without LiDFOB. The interaction of Cl^-–Cl^- was weakened in the presence of $DFOB^-$.

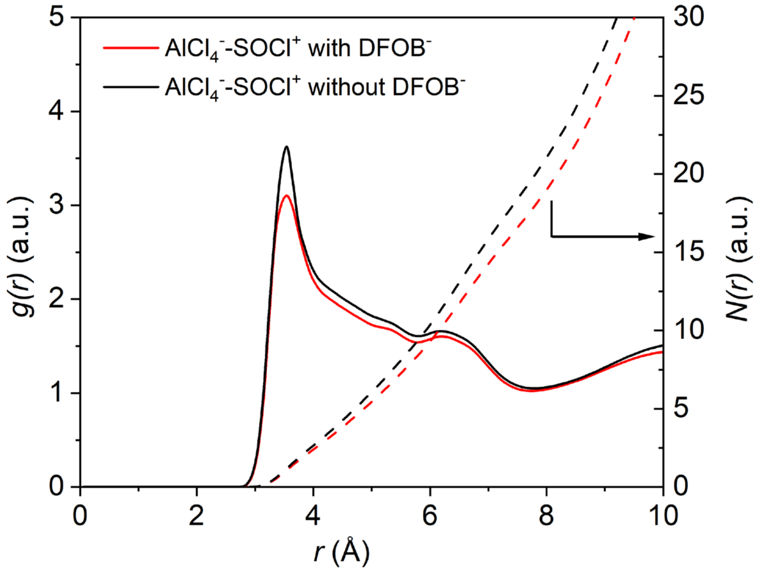

157

158 **Supplementary Fig. 10 RDFs of $AlCl_4^-$–$SOCl^+$ in CALS electrolyte with and without**
159 **LiDFOB.** The interaction of $AlCl_4^-$–$SOCl^+$ was weakened in the presence of $DFOB^-$.

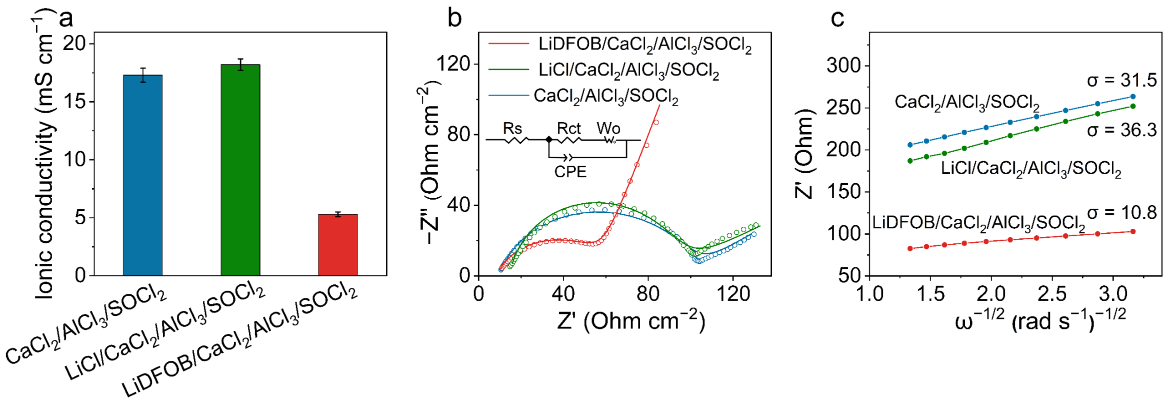

Supplementary Fig. 11 Comparison of the ionic conductivities and EIS results of $CaCl_2/AlCl_3/SOCl_2$, $LiCl/CaCl_2/AlCl_3/SOCl_2$ and $LiDFOB/CaCl_2/AlCl_3/SOCl_2$ electrolytes. a, Ionic conductivities of $CaCl_2/AlCl_3/SOCl_2$, $LiCl/CaCl_2/AlCl_3/SOCl_2$ and $LiDFOB/CaCl_2/AlCl_3/SOCl_2$ electrolytes. **b,** Nyquist plots of the as-prepared Ca/Cl_2 batteries using $CaCl_2/AlCl_3/SOCl_2$, $LiCl/CaCl_2/AlCl_3/SOCl_2$ and $LiDFOB/CaCl_2/AlCl_3/SOCl_2$ electrolytes. The inset showed the corresponding equivalent circuit. **c,** Linear correlations between Z' and the square root of frequency ($\omega^{-1/2}$) in the low-frequency regions of the EIS plots for these batteries. The concentrations of $AlCl_3$ and $CaCl_2$ were 6 M and 1.2 M, respectively. The concentrations of LiCl and LiDFOB were both 1.3 M.

The ion diffusion coefficient (D) is inversely proportional to σ^2 ($D \sim 1/\sigma^2$), and the calculation of σ follows equation (S2) as below:

$$Z' = \sigma\omega^{-1/2} + R_s + R_{ct} \qquad \text{(S2)}$$

where Z' is the real part of impedance (Z). $\omega^{-1/2}$ is the square root of angular frequency in the low-frequency region. σ is the Warburg factor, and also represents the slope of the straight line in the graph.

The ion diffusion coefficient was calculated according to equation (S3):

$$D = R^2T^2/2A^2n^4F^4C^2\sigma^2 \qquad \text{(S3)}$$

where D is the diffusion coefficient, R is the gas constant, T is the absolute temperature, A is the effective area of electrode, n is the number of transferred electrons, F is the Faraday constant, C is the concentration of ions, and σ is the Warburg factor that is related to Z' according to equation (S3).

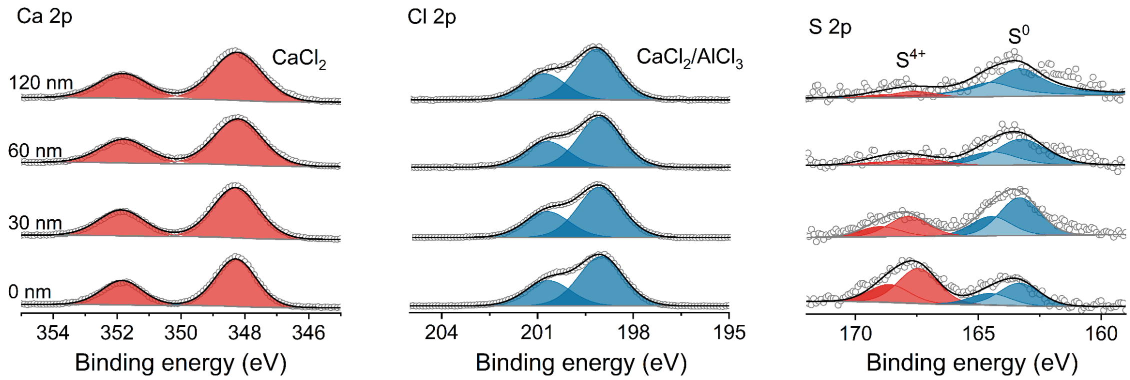

Supplementary Fig. 12 XPS profiles of the graphite cathode after the first discharge. High-resolution XPS spectra of Ca 2p, Cl 2p, and S 2p of the graphite cathode after the first discharge of the battery using CALS electrolyte. Current density, 100 mA g^{-1}. The XPS depth profiling verified the presence of $CaCl_2$, SO_2 (S^{4+}) and sulfur (S^0) on the graphite cathode. $CaCl_2$ was uniformly distributed throughout the sputter depth, and SO_2 was mainly distributed on the surface of the graphite cathode.

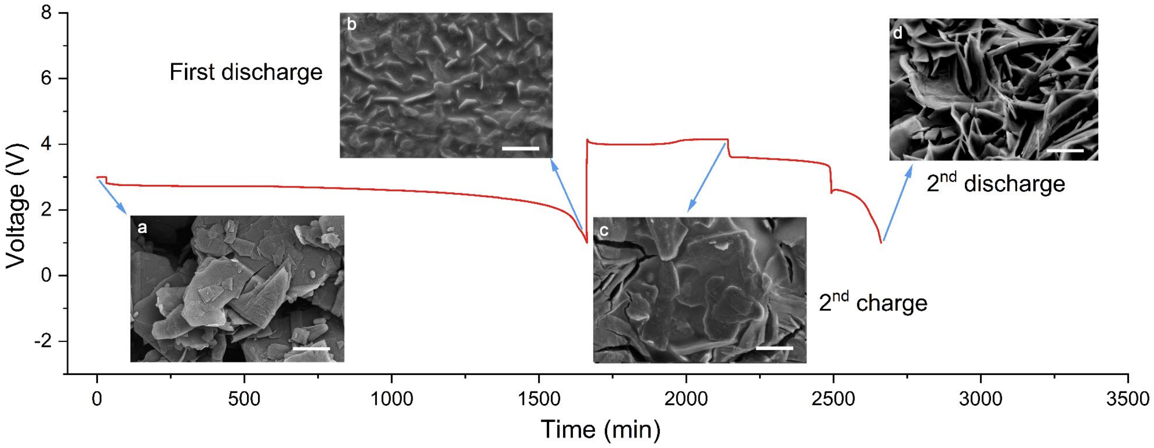

Supplementary Fig. 13. SEM images of the graphite cathodes at different charge/discharge states. **a**, Pristine, **b**, 1st discharge, **c**, 2nd charge, **d**, 2nd discharge. Current density, 100 mA g^{-1}. Specific charge capacity, 800 mAh g^{-1}. Scale bars in **a-d**, 1 μm.

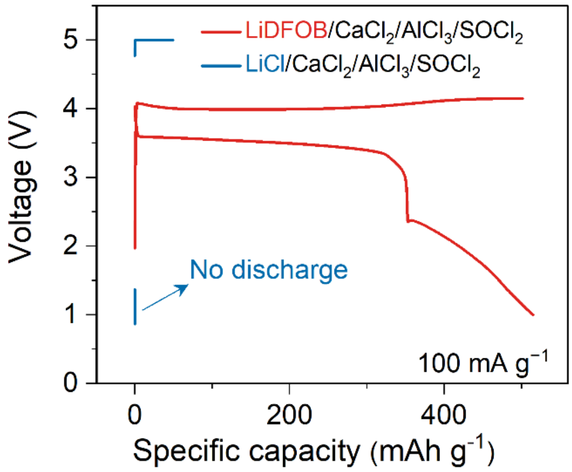

Supplementary Fig. 14. **Galvanostatic charge–discharge curves of the Ca/Cl_2 batteries using the LiDFOB/$CaCl_2$/$AlCl_3$/$SOCl_2$ (CALS) and LiCl/$CaCl_2$/$AlCl_3$/$SOCl_2$ electrolytes.** The specific charge capacity and current density are 500 mAh g^{-1} and 100 mA g^{-1}, respectively.

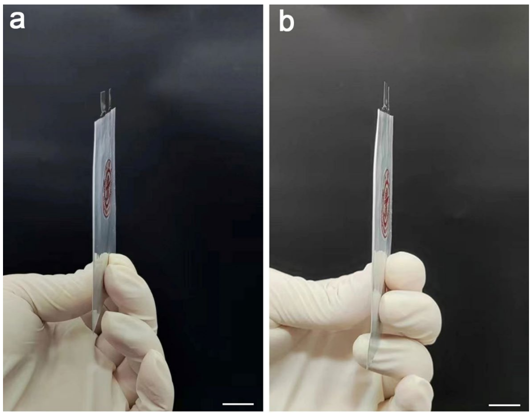

Supplementary Fig. 15. **Photographs of a rechargeable Ca/Cl_2 pouch cell at different states.** Ca/Cl_2 pouch cell using the CALS electrolyte before (**a**) and after charging (**b**). Scale bars, 1 cm.

When fully charging the Ca/Cl_2 pouch cell, no obvious volume change had been observed, suggesting that the Cl_2 might be adsorbed/trapped by graphite, which avoid the severe increase of the internal vapor pressure of the obtained battery.

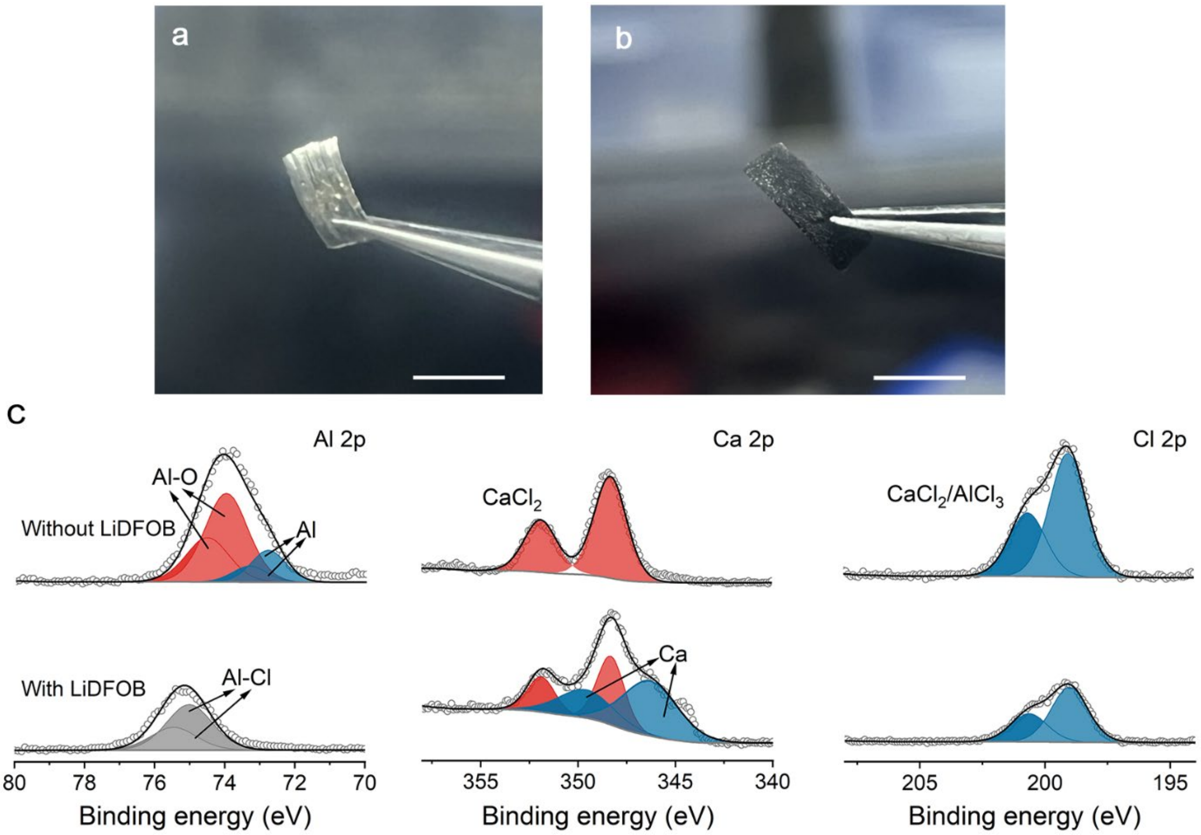

Supplementary Fig. 16 Characterization of Ca metal anodes immersing in different electrolytes. a, **b**, The Ca metal foils after immersing in the CALS electrolytes with and without LiDFOB for 2 h, respectively. Scale bars, 1 cm. **c,** High-resolution Al 2p, Ca 2p, and Cl 2p spectra of XPS profile of the Ca metal foils immersed in the CALS electrolytes with and without LiDFOB for 2 h.

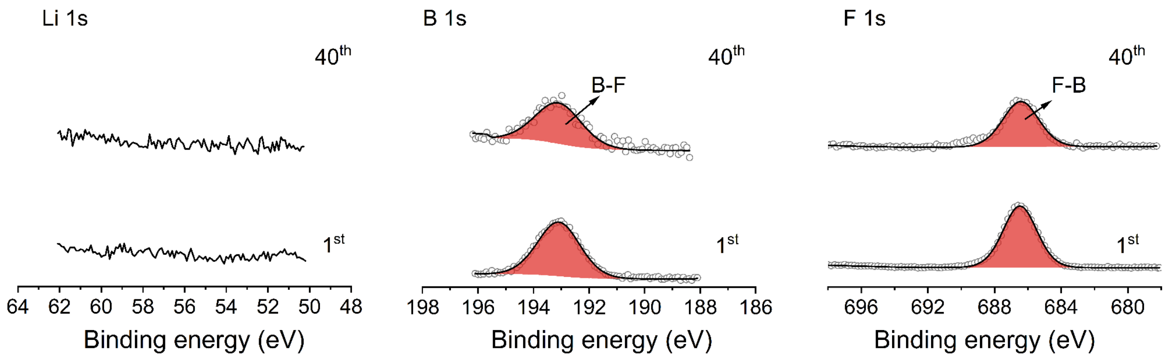

Supplementary Fig. 17 XPS analysis of Ca anodes after 1st and 40th cycles. High-resolution XPS spectra of Li 1s, B 1s, and F 1s of the charged anodes of the rechargeable Ca/Cl_2 battery using CALS electrolyte after 1st and 40th cycles.

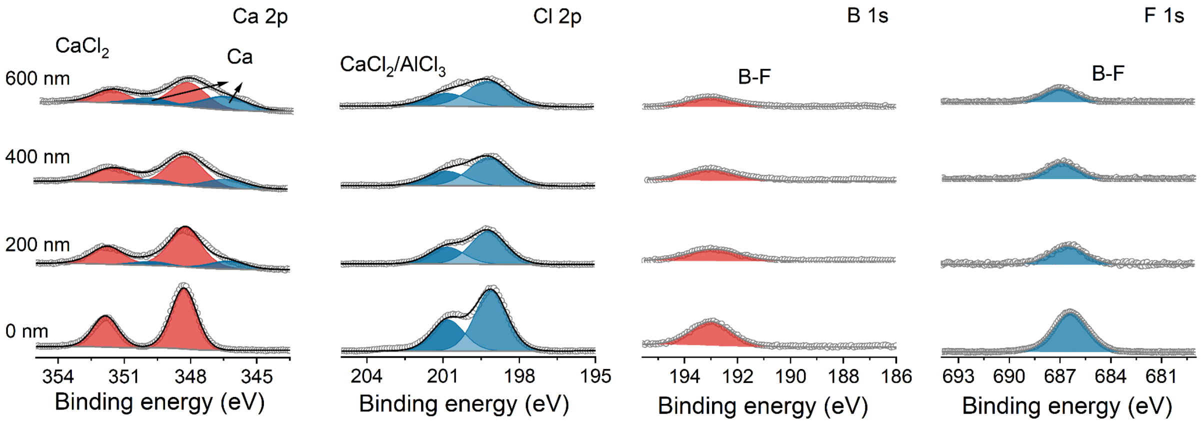

Supplementary Fig. 18 XPS analysis of the Ca metal anode after the first discharge. High-resolution XPS spectra of Ca 2p, Cl 2p, B 1s, and F 1s spectra of XPS depth profile of the Ca metal anode in a first discharged Ca/Cl_2 battery using CALS electrolyte. Current density 100 mA g^{-1}.

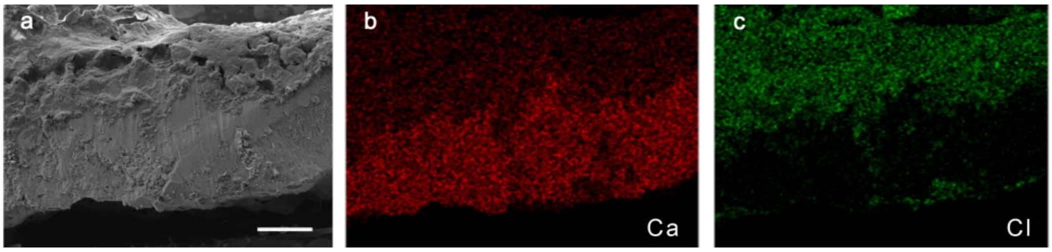

Supplementary Fig. 19 Characterization of Ca metal anode in a fully charged Ca/Cl_2 battery using CALS electrolyte. a, Cross-section SEM image. **b**, EDS mappings of Ca and Cl elements. The battery was cycled for 14 cycles prior to characterization. Specific charge capacity, 400 mAh g^{-1}. Current density, 100 mA g^{-1}. Scale bar in **a**, 100 μm.

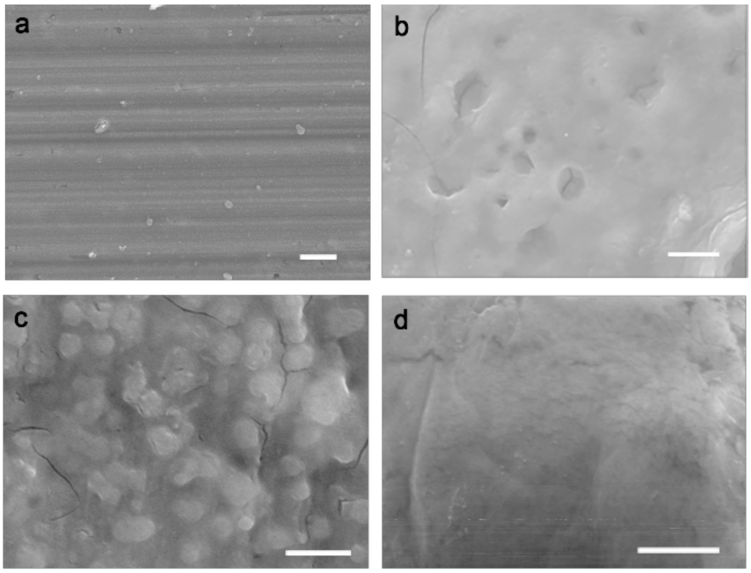

Supplementary Fig. 20 SEM images of Ca metal anodes in rechargeable Ca/Cl_2 batteries at various charge/discharge states. **a**, Pristine. **b**, 1st discharge. **c**, 2nd charge, **d**, 2nd discharge. Scale bars, 5 μm. Current density, 100 mA g^{-1}.

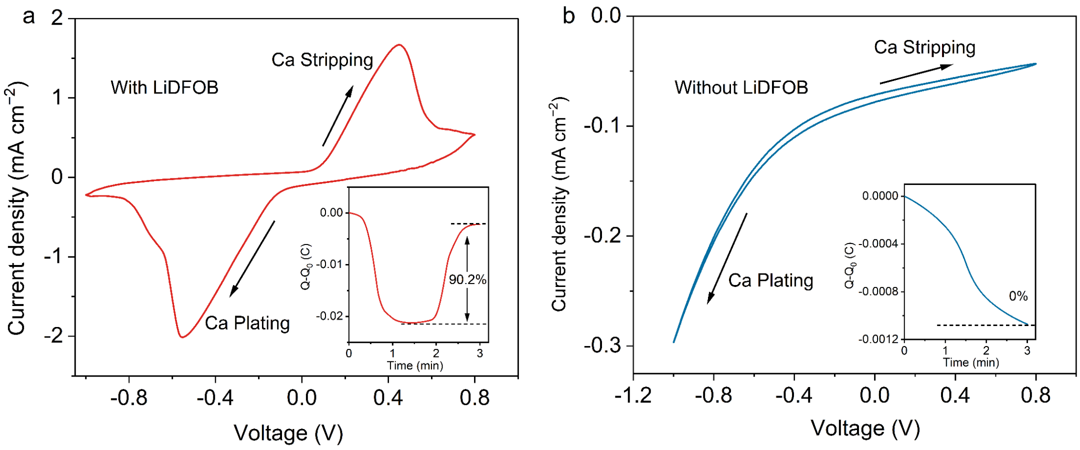

Supplementary Fig. 21 CV curves of Ca/Au cells using different electrolytes. a, CALS electrolyte. **a**, CALS electrolyte without LiDFOB. Scan rate, 20 mV s^{-1}. The insets showed the Coulombic efficiencies of the Ca plating and stripping process.

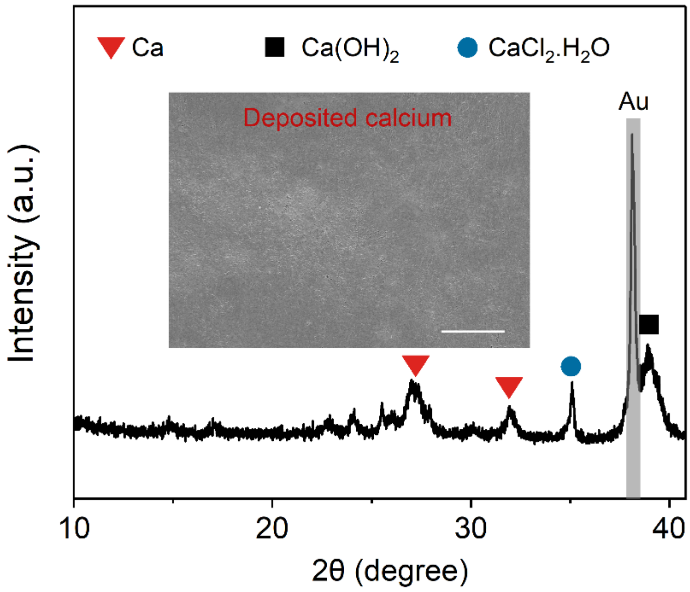

Supplementary Fig. 22 XRD profile and SEM image of the deposited Ca metal. XRD profile of the deposited Ca metal on Au. The inset showed the SEM image of the deposited Ca metal. Scale bar, 20 μm.

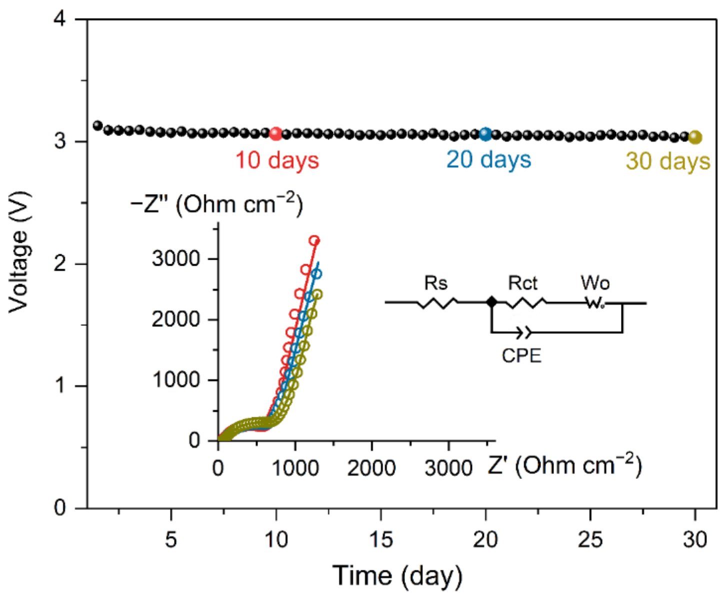

Supplementary Fig. 23 Retention performance of the rechargeable Ca/Cl_2 battery. Variation of the open-circuit voltage of a Ca/Cl_2 battery during 30-day retention. The EIS analysis of the Ca/Cl_2 battery after retention for 10, 20, and 30 days showed minor differences in charge transfer resistance (R_{ct}) based on the equivalent circuit, which indicated the excellent retention performance.

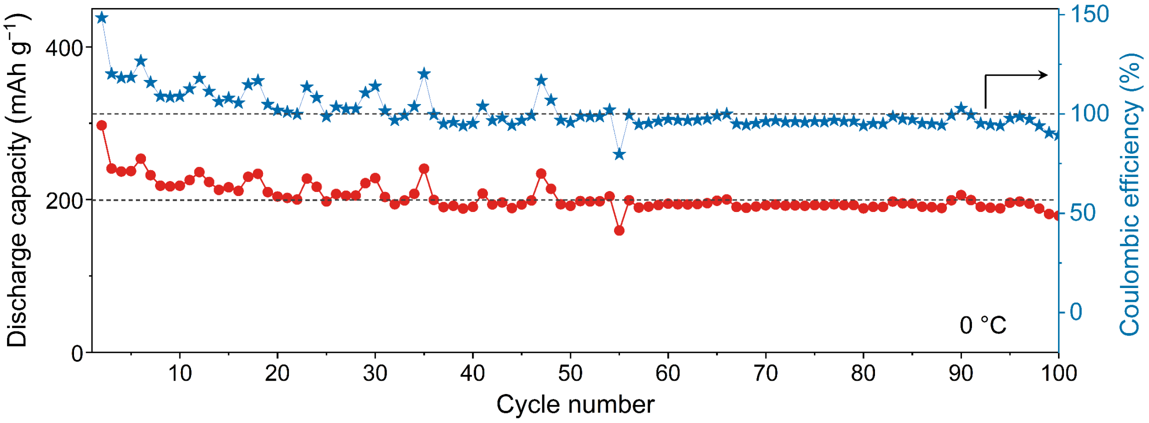

Supplementary Fig. 24 Low-temperature performance of the rechargeable Ca/Cl_2 battery. Cyclic performance of the Ca/Cl_2 battery at 0 °C. The specific charge capacity and current density were 200 mAh g^{-1} and 100 mA g^{-1}, respectively.

Supplementary Table 1. Stoichiometric ratio of different ions in the CALS and CAS simulation boxes.

Ion species	$AlCl_4^-$	$DFOB^-$	Li^+	Ca^{2+}	Cl^-	$SOCl^+$	$SOCl_2$
CALS system	1100	238	238	220	440	1100	1411
CAS system	1100	−	−	220	440	1100	1411

Supplementary Table 2. The average atomic composition in the solvation shell of Ca^{2+} ions of CALS and CAS electrolyte systems.

Electrolytes	$AlCl_4^-$	Cl^-	$DFOB^-$
CALS system	3.0	2.4	2.2
CAS system	1.9	5.2	−

Supplementary References

1 Mosier-Boss, P., *et al.* Raman and infrared spectroscopy of the $AlCl_3$-$SOCl_2$ system. *J. Chem. Soc., Faraday Trans. 1.* **85**, 11−21 (1989).

2 Hess, B., Kutzner, C., Spoel, D. & Lindahl, E. Gromacs 4: Algorithms for highly efficient, load-balanced, and scalable molecular simulation. *J. Chem. Theory Comput.* **4**, 435−447 (2008).

3 Case D., Ben-Shalom I., Brozell S., Ceruti D., Cheatham T., Cruzeiro V., *et al*. Amber 18. 2018. *University of California, San Francisco.*

4 Wang, J., Wolf, R. M., Caldwell, J. W., Kollman, P. A., and Case, D. A. Development and testing of a general amber force field. *J. Comput. Chem.* **25**,1157−74 (2004).

5 Bayly, C. I., Cieplak, P., Cornell, W. & Kollman, P. A. A well-behaved electrostatic potential based method using charge restraints for deriving atomic charges: the RESP model. *J. Phys. Chem.* **97**,10269−10280 (2002).

6 Doherty, B., Zhong, X., Gathiaka, S., Li, B. & Acevedo, O. Revisiting OPLS force field parameters for ionic liquid simulations. *J. Chem. Theory Comput.* **13**, 6131−6145 (2017).

7 Parrinello, M. & Rahman, A. Crystal structure and pair potentials: A molecular-dynamics study. *Phys. Rev. Lett.* **45**, 1196−1199 (1980).

8 Holoubek, J. *et al*. Tailoring electrolyte solvation for Li metal batteries cycled at ultra-low temperature. *Nat. Energy* **6**, 303–313 (2021).

9 Hess, B., Bekker, H., Berendsen, H. J. C. & Fraaije, J. LINCS: A linear constraint solver for molecular simulations. *J. Comput. Chem.* **18**, 1463−1472 (1997).

10 Humphrey, W., Dalke, A. & Schulten, K. VMD: Visual molecular dynamics. *J. Mol. Graph.* **14**, 33−38 (1996).

11 Essmann, U., Perera, L., Berkowitz, M., Darden, T., Lee, H. & Pedersen, L. G. A smooth particle mesh Ewald method. *J. Chem. Phys.* **103**, 8577−8593 (1995).

12 Hou S. *et al.* Solvation sheath reorganization enables divalent metal batteries with fast interfacial charge transfer kinetics. *Science* **374**, 172−178 (2021).

13 Li Z. *et al.* Rechargeable calcium–sulfur batteries enabled by an efficient borate-based electrolyte. *Small* **16**, 2001806 (2020).

14 Tohru S, Yuichi K & Yoko H,. Coupling of nitroxyl radical as an electrochemical charging catalyst and ionic liquid for calcium plating/stripping toward a rechargeable calcium–oxygen battery. *J. Mater. Chem. A* **5**, 13212 (2017).

15 Zheng, J. *et al.* Directed self-assembly of herbal small molecules into sustained release hydrogels for treating neural inflammation. *Nat. Commun.***10**, 1604 (2019).

16 Judith M., César S., & David D . Release of small bioactive molecules from physical gels. *Chem. Soc. Rev.* **47**, 1484-1515 (2018).
